# Ameliorative effect of melatonin improves drought tolerance by regulating growth, photosynthetic traits and leaf ultrastructure of maize seedlings

**DOI:** 10.1186/s12870-021-03160-w

**Published:** 2021-08-12

**Authors:** Shakeel Ahmad, Ihsan Muhammad, Guo Yun Wang, Muhammad Zeeshan, Li Yang, Izhar Ali, Xun Bo Zhou

**Affiliations:** Guangxi Colleges and Universities Key Laboratory of Crop Cultivation and Tillage, Agricultural College of Guangxi University, Nanning, 530004 China

**Keywords:** Melatonin, Leaf ultrastructure, Antioxidant enzymes, Drought stress, Maize

## Abstract

**Background:**

Melatonin is considered a potential plant growth regulator to enhance the growth of plants and increase tolerance to various abiotic stresses. Nevertheless, melatonin’s role in mediating stress response in different plant species and growth cycles still needs to be explored. This study was conducted to understand the impact of different melatonin concentrations (0, 50, 100, and 150 μM) applied as a soil drench to maize seedling under drought stress conditions. A decreased irrigation approach based on watering was exposed to maize seedling after drought stress was applied at 40–45% of field capacity.

**Results:**

The results showed that drought stress negatively affected the growth behavior of maize seedlings, such as reduced biomass accumulation, decreased photosynthetic pigments, and enhanced the malondialdehyde and reactive oxygen species (ROS). However, melatonin application enhanced plant growth; alleviated ROS-induced oxidative damages by increasing the photosynthetic pigments, antioxidant enzyme activities, relative water content, and osmo-protectants of maize seedlings.

**Conclusions:**

Melatonin treatment also enhanced the stomatal traits, such as stomatal length, width, area, and the number of pores under drought stress conditions. Our data suggested that 100 μM melatonin application as soil drenching could provide a valuable foundation for improving plant tolerance to drought stress conditions.

## Background

Drought is one of the abiotic stresses that limit plant growth and production, directly affecting the yield of crops [1]. China has been described as a drought-affected country because of the difference in weekly, annual and inter-annual precipitation and its temperature [2]. In the last decade, droughts were an inherent famine phenomenon resulting in an average grain loss of 39.2 billion kg in China, causing an economic loss of 14.7% to the world GDP [3]. Drought-induced osmotic stress mainly decreases plant water absorption; it triggers physiological and biochemical imbalances leading to stomatal closure, decreased photosynthesis and transpiration, decreased cell capacity, and increased oxidative stress [4–6]. However, plants have established an antioxidant defense system under environmental stress [7]. However, the deterioration in the antioxidant protection mechanism can occur when the stress level exceeds the plant protection capability, and the excessive accumulation of ROS can result in protein, lipid, and DNA oxidation [8]. The plants developed defense mechanisms containing enzymatic antioxidants such as superoxide dismutase (SOD), catalase (CAT), peroxidase (POD), ascorbate peroxidase (APX), and ascorbate glutathione (AsA-GSH) cyclic enzymes, and non-enzymatic antioxidants such as glutathione (GSH), ascorbate (ASA), and carotenoids [7, 9]. Reactive oxygen species mainly include superoxide (O_2_), hydroxyl (OH^•^) and hydrogen peroxide (H_2_O_2_) in plants. Furthermore, by altering the primary and secondary metabolites, plants can also accumulate soluble sugar and proline in cells, thus encouraging water retention to be adopted in the dry environment [10–12]. Therefore, numerous studies have been carried out to enhance plant tolerance and mitigate the adverse effects of various environmental stresses, including drought stress.

The use of phytohormones to increase adaptability and protect plants from adverse environmental conditions is one of the innovative strategies [13]. Various plant phytohormones have beneficial effects on different plant physiological processes [14, 15]. As a major hormone and natural compound of indole-amine, melatonin (N-acetyl-5-methoxytryptamine) modulates many physiological and biochemical processes that may positively affect plant development [16]. Melatonin has gained tremendous interest in the scientific community because of its wide distribution in the biological kingdom and its possible role in many plant physiological processes. While melatonin has been reported to promote plant growth and protection against various abiotic stresses in different crops [17]. Melatonin pretreatment can improve stress tolerance and inhibit ROS-induced oxidative damage by controlling stress response gene expression. Melatonin protects plants from the harmful effects of drought-induced oxidative stress by increasing the efficiency of ROS scavenging capacity [18]. In addition, as a non-toxic, biodegradable molecule, melatonin is strongly recommended to encourage environmentally sustainable crop production.

Although the function of melatonin in crop plant stress tolerance has been previously reported in various studies. However, the impact of melatonin as a soil drenching application and its effect on root growth and leaf ultrastructure of maize seedlings under drought stress is still unclear. Therefore, melatonin-mediated physiological, biochemical, and growth changes in maize seedlings exposed to drought stress were investigated in our current research. We also examined the effect of plant growth, photosynthetic capacity, oxidative system, osmolytes accumulation, and the leaf stomatal ultrastructure traits of maize seedlings under drought stress conditions. This research would lead to elucidating the mitigating effects of melatonin on oxidative damage caused by drought stress.

## Results

### Effect of melatonin on root traits, biomass accumulation, and RWC (%) of maize seedling

Drought stress had a significant inhibitory effect on the overall growth of maize plants, but the application of melatonin significantly increased the growth characteristics of maize seedlings. Under control drought stress (CKD), fresh weight (FW) and dry weight (DW) root biomass of maize seedlings were significantly lower compared to well water conditions (CKW). Table 1 showed that the adverse effects of drought stress on biomass accumulation were greatly reduced and steadily increased with increasing melatonin concentration. Melatonin application MT2 treatment improved root FW and DW by 24.04 and 46.97% of maize seedlings and total above dry matter by 28.85% compared to CKD. In addition, the root to shoot ratio of maize seedling was higher in treatment MT2 by 14.14% compared with control CKD, indicating that melatonin treatments promoted root growth more than shoot growth. The application of melatonin as soil drench promoted root elongation of maize seedlings and was significantly higher by 29.08% in treatment MT2. The results show that melatonin application under drought stress conditions triggered an increase in root length. However, the reduction in root length was observed in CKD treatment by 27.3% compared to CKW and other melatonin treatment plants, respectively.

**Table 1:** Effect of melatonin on root characteristics, biomass, and relative water content (RWC %) of maize seedling under drought stress condition

Treatments	Root Fresh Weight (g)	Root Dry Weight (g)	Root Diameter (mm)	Root Length (cm plant^−1^)	Above Dry Matter (g plant^−1^)	Root/Shoot Ratio	RWC (%)
CKW	10.21 ± 0.65a	0.96 ± 0.07a	1.66 ± 0.05a	911.01 ± 18.76a	22.61 ± 1.22a	0.043	97.15 ± 2.52a
CKD	7.76 ± 0.23d	0.61 ± 0.03c	1.23 ± 0.03d	662.33 ± 32.51e	14.77 ± 0.65d	0.041	73.96 ± 0.99d
MT1	8.89 ± 0.32c	0.70 ± 0.50c	1.29 ± 0.04d	718.03 ± 21.75d	16.83 ± 0.68c	0.042	80.62 ± 1.48c
MT2	9.62 ± 0.34b	0.89 ± 0.03ab	1.50 ± 0.03b	854.95 ± 27.12b	19.03 ± 0.95b	0.047	89.72 ± 2.61b
MT3	9.07 ± 0.29bc	0.81 ± 0.60b	1.38 ± 0.02c	808.47 ± 15.13c	18.96 ± 0.77b	0.043	86.57 ± 1.32b

Melatonin application as a soil drenching: CKW; indicate Melatonin 0 μM + Control well water, CKD; Melatonin 0 μM + Control drought stress, MT1; Melatonin 50 μM + drought stress, MT2; Melatonin 100 μM + drought stress; MT3; Melatonin 150 μM + drought stress. Data are shown as the mean ± S.D. each treatment replicated three times (*n* = 3). Different letters indicate a significant difference among the treatments at *P* ≤ 0.05

Similarly, average root diameter showed a steady increase in melatonin-treated plants compared to CKD. We observed that melatonin-treated seedling had a thicker root diameter and was greater by 22.18% in MT2 than CKD (Table 1). Higher doses of melatonin had inhibitory effects on maize seedlings under stress conditions, suggesting that we should optimize melatonin concentrations for crop growth.

Relative water content (RWC) is the key indicator that shows the plant’s survival capability and leaf water status. The CKD significantly reduced the RWC compared to CKW. The leaf RWC was markedly greater in all melatonin treatments than in CKD. Under drought stress conditions, the most significant increase in RWC was observed in melatonin treatment MT2 by 21.30% compared to CKD (Table 1).

### Effect of melatonin on gas exchange and chlorophyll carotenoid

**Fig. 1:**
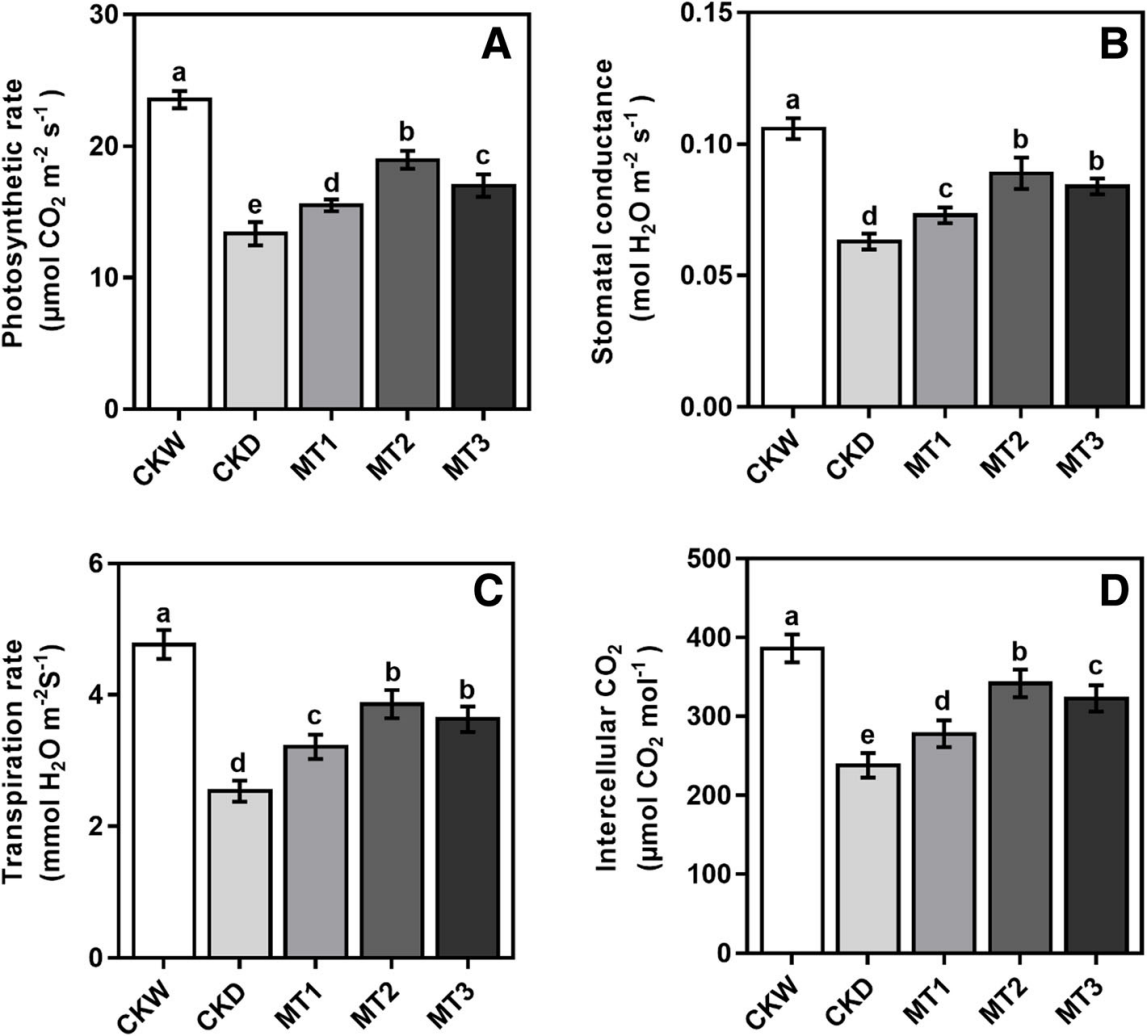
Effects of melatonin on net photosynthetic rate, transpiration rate, stomatal conductance, and intercellular CO_2_ of maize seedlings under drought stress. CKW; indicate Melatonin 0 μM + Control well water, CKD; Melatonin 0 μM + Control drought stress, MT1; Melatonin 50 μM + drought stress, MT2; Melatonin 100 μM + drought stress; MT3; Melatonin 150 μM + drought stress. Vertical bars represent ± S.D. (*n*=3). Different small letters indicate significant differences as determined by the LSD test (*p* ≤ 0.05)

**Fig. 2:**
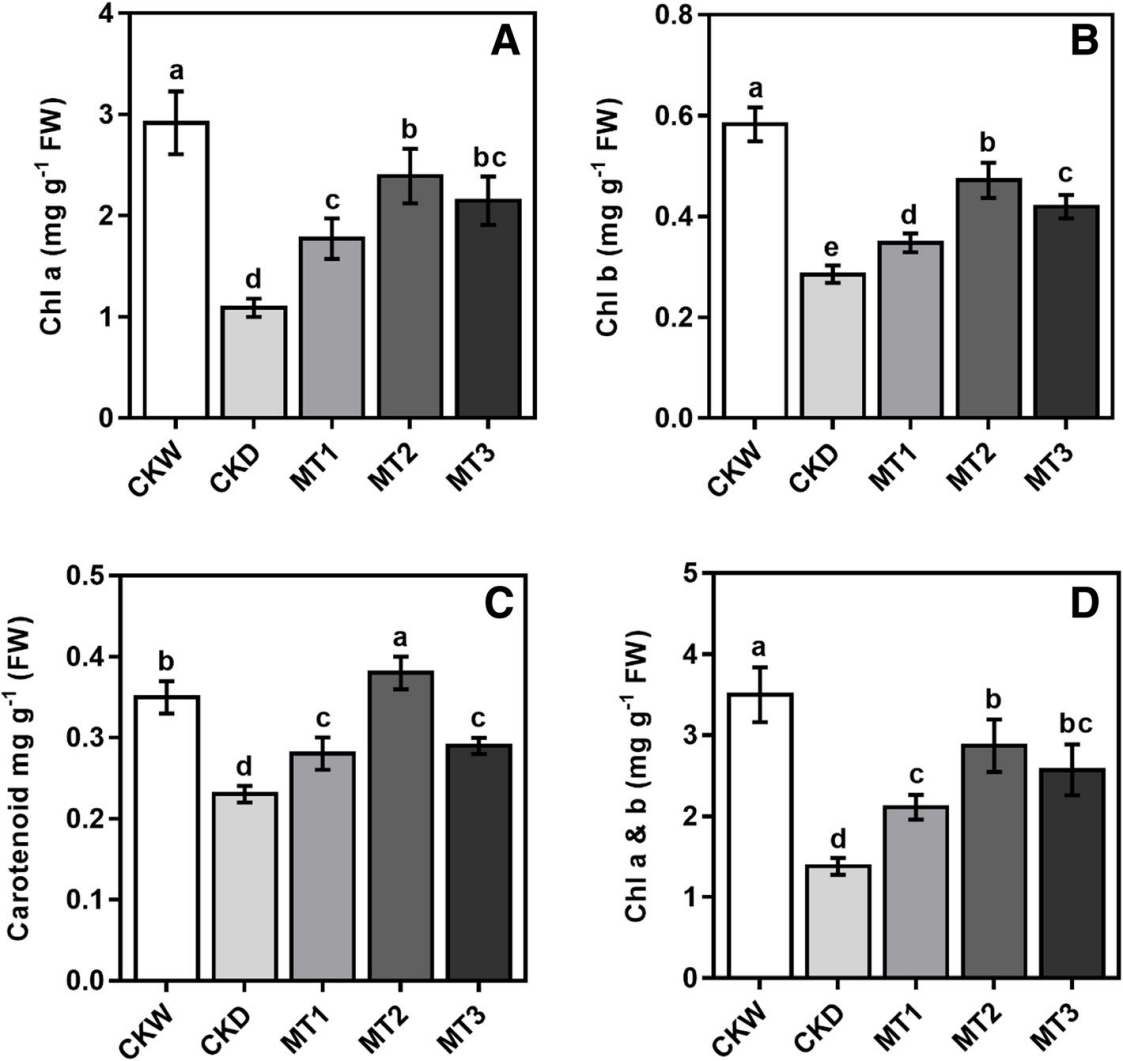
Effects of melatonin on Chlorophyll a, chlorophyll b, carotenoid, and chlorophyll a & b (mg g^-1^ FW) contents of maize seedlings under drought stress. The names of the treatments are the same as in (Fig. 1). Different lowercase letters indicate significant differences as determined by the LSD test (*p* ≤ 0.05)

A severe decrease in the photosynthetic gas exchange of maize seedlings was observed under drought stress conditions. Though, exogenous application of melatonin ameliorated the stress-induced reduction in gas exchange parameters. Our results showed that melatonin treatment MT2 significantly increased photosynthetic rate, stomatal conductance, transpiration rate, and intercellular CO_2_ by 42.01, 41.56, 52.10, and 43.51% as compared with the CKD (Fig. 1). Our results showed obvious indications of drought stress on chlorophyll a, b, carotenoids concentration, and total chlorophyll content compared to CKD. On the contrary, plants in all melatonin treatments exhibited noticeable diminution of the antagonistic effects of drought stress. However, melatonin application had a promising effect on increasing the chlorophyll and carotenoid contents under drought stress conditions. The results showed that higher melatonin treatment MT3 employed a negative influence on the chlorophyll contents and provoked a steady decline compared with MT2 treatments. As shown in Fig. 2, the melatonin treatment MT2 enhanced the chlorophyll-a content by 119.16%, chlorophyll-b by 65. 29%, carotenoid by 63.27%, and total chlorophyll contents by 107.96% compared to CKD.

### Effect of melatonin on SEM imaging of stomatal traits and behavior

**Fig. 3:**
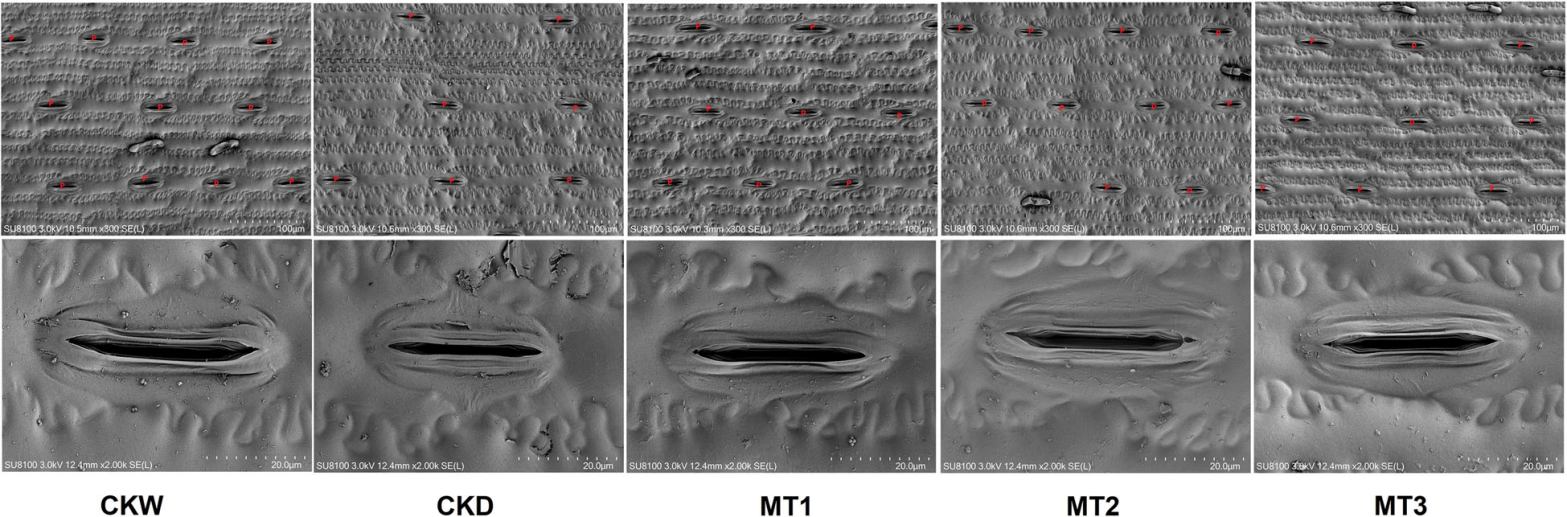
Effect of melatonin application on stomatal length (μM), stomatal width (μM), stomatal area (μm^2^), and the number of pores under drought stress condition. The treatments names are the same as those described in (Fig. 1). For stomatal number X300 magnification, scale bars =100 μm, while for stomatal length (μM), stomatal width (μM), stomatal area (μm^2^) X2000 magnification, scale bars =20 μm

**Fig. 4:**
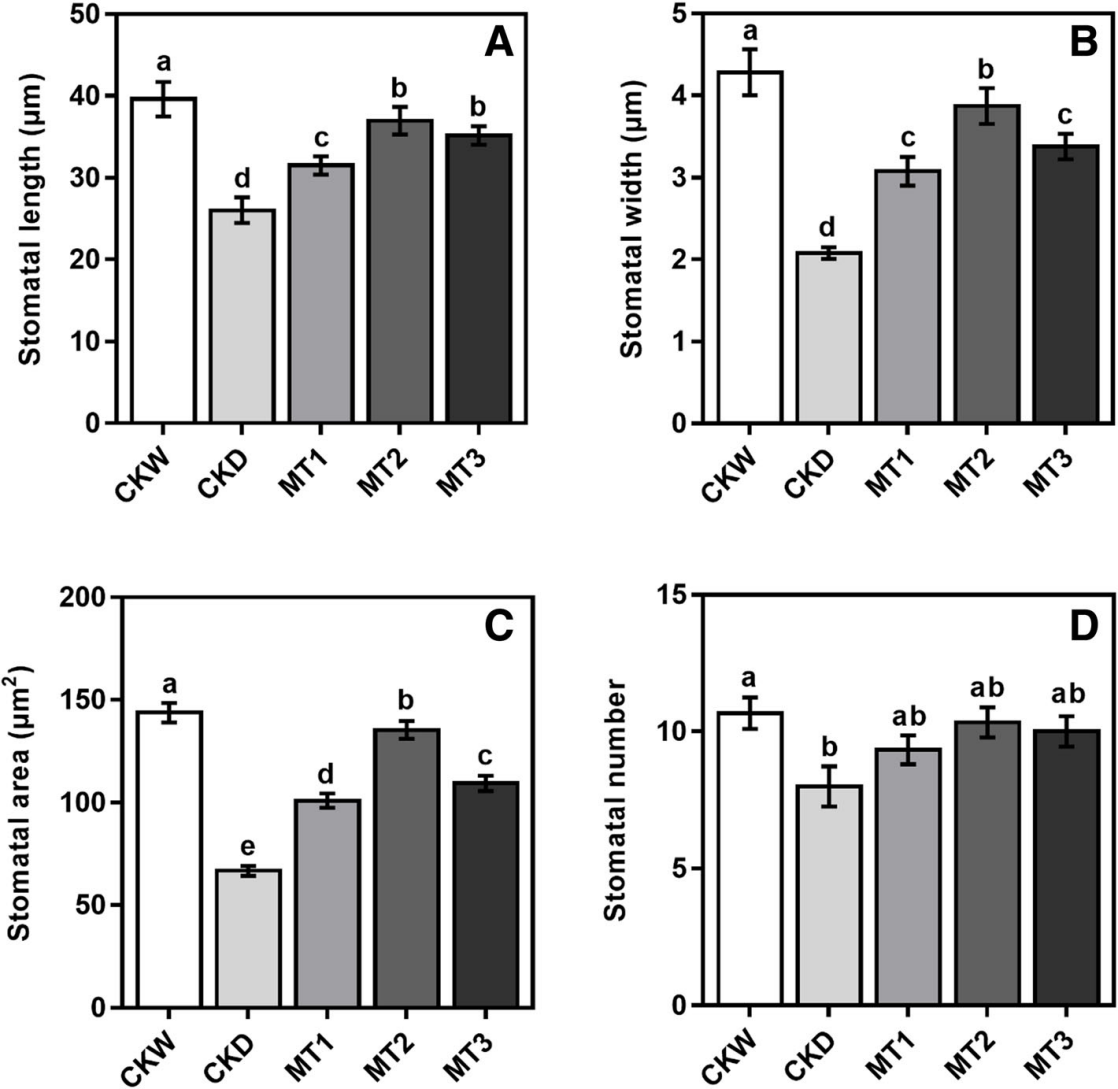
Effect of melatonin application on stomatal length (μM), stomatal width (μM), stomatal area (μm^2^), and the number of pores of maize seedling under drought stress condition. The names of the treatments are the same as in (Fig. 1). Different lowercase letters indicate significant differences as determined by the LSD test (*p* ≤ 0.05)

Stomatal parameters and simple structures were altered by drought stress. Morpho-physiological and biochemical trait analysis showed that exogenous melatonin application significantly improved stomatal length, stomatal width, stomatal area, and the number of pores under drought stress conditions (Fig. 3). In plants, water vapor and air exchange stomata are the important passages. Drought stress significantly reduced the stomatal length, width, area, and the number of pores by 34.27, 51.52, 53.62, and 25.01% compared with CKW. However, our results showed that melatonin treatment MT2 increased the stomatal length, width, area, 42.07, 86.52, 102.84%, and the number of pores significantly similar in melatonin treated plants as compared to CKD (Fig. 4).

### Effect of melatonin on soluble sugar and proline content

**Fig. 5:**
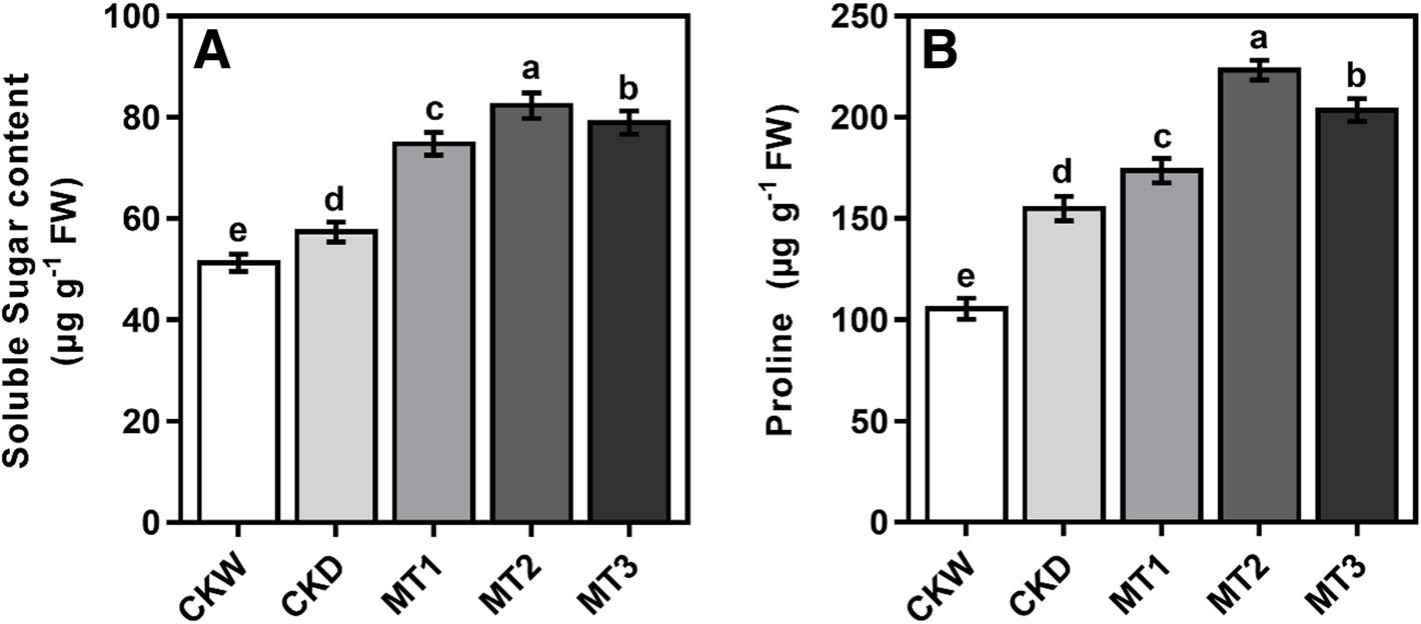
Effect of melatonin application on soluble sugar and proline content of maize seedling under drought stress condition. The names of the treatments are the same as in (Fig. 1). Different lowercase letters indicate significant differences as determined by the LSD test (*p* ≤ 0.05)

Soluble sugar and proline contents are the important osmo-protectants which enable the plants to tolerate oxidative damages under abiotic stress condition. Compared with the CKW, soluble sugars and proline, were significantly improved after the imposition of drought stress. Exogenous melatonin application further enhanced the levels of osmotic regulators under drought stress conditions (Fig. 5). Remarkably, soluble sugar and proline contents increased in all melatonin treatments under drought stress compared to CKW. The results showed that melatonin treatment (MT2) increased the soluble sugar and proline content by 43.55 and 50.55%, but the soluble sugar was significantly similar to MT3.

### Effect of melatonin on soluble protein, MDA and ROS

**Fig. 6:**
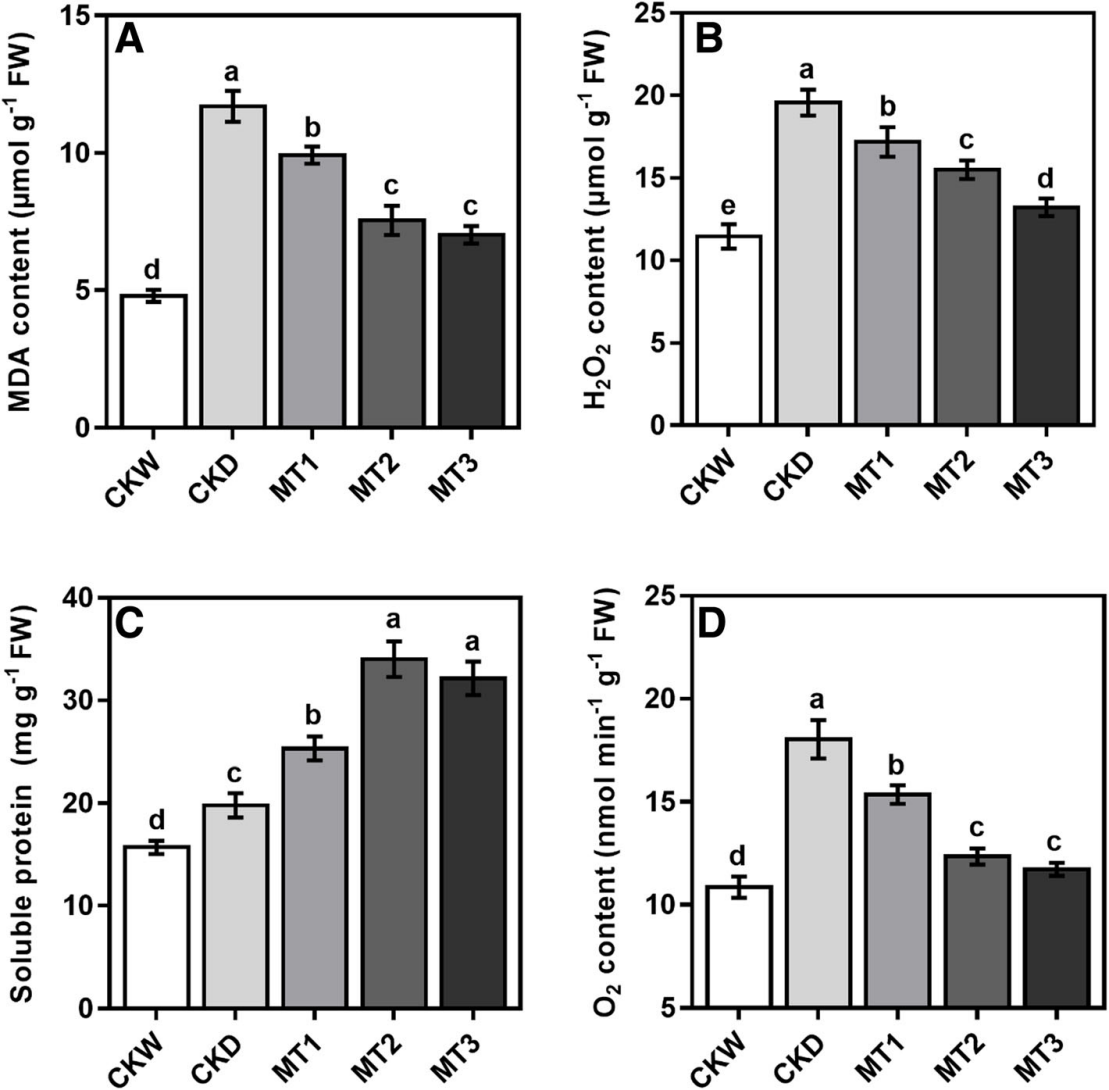
Effect of melatonin application on MDA, soluble protein, H_2_O_2_ and O_2_ contents of maize seedling under drought stress conditions. The names of the treatments are the same as in (Fig. 1). Different lowercase letters indicate significant differences as determined by the LSD test (*p* ≤ 0.05)

The soluble protein content was increased after drought stress, demonstrating the biochemical modification of maize seedlings to drought stress conditions. The soluble protein content was further increased in melatonin treatment at a variable degree. As shown in Fig. 6, soluble protein content was significantly higher by 71.86% in melatonin treatment MT2 than CKW. Consequently, the initiation of drought stress triggers a large enhancement in MDA accumulation as compared to CKW. Our results showed that the application of melatonin significantly decreases malondialdehyde (MDA) accumulation under drought stress conditions by increasing the concentration of melatonin (Fig. 6). The MDA content of the treatments MT1, MT2, MT3 decreased by 15.05, 35.40, and 39.93% and was greater by 142.30% in CKW than CKD. Compared with untreated CKW, the least MDA contents were observed for MT3 treatments but significantly similar to MT2.

The H_2_O_2_ and O_2_ are ROS produced by plant cells more prominently under abiotic stress conditions. In the present study, we also assessed the content of H_2_O_2_ and O_2_ in the leaves to determine the effect of melatonin on ameliorating oxidative stress caused by drought. Drought stress significantly enhanced the H_2_O_2_ and O_2_ as compared with CKW. The generation of H_2_O_2_ and O_2_ has been steadily attenuated, regardless of the melatonin application process, with increased melatonin concentrations under drought stress conditions. Our findings showed that the use of melatonin application substantially decreased ROS by increasing the concentrations of melatonin. The results showed that melatonin treatments MT1, MT2, and MT3 reduced the H_2_O_2_ content by 11.31, 19.21, and 31.48%, while the O_2_ contents by 14.80, 31.50, and 33.31% as compared to drought stress control CKD (Fig. 6).

### Effect of melatonin on the activities of antioxidant enzymes

**Fig. 7:**
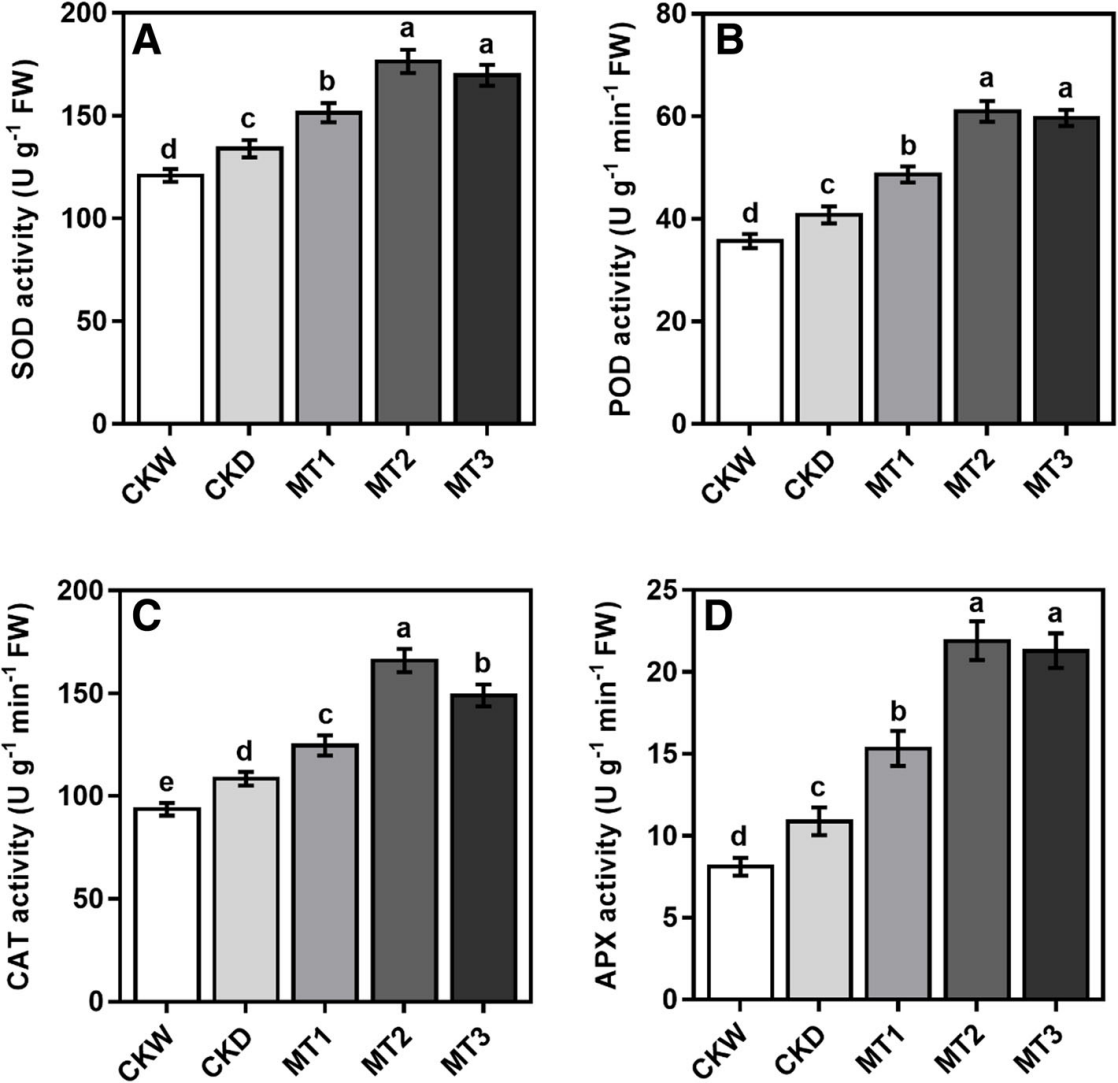
Effect of melatonin application on antioxidant enzymes activities (SOD, POD, CAT and APX) of maize seedling under drought stress conditions. The names of the treatments are the same as in (Fig. 1). Different lowercase letters indicate significant differences as determined by the LSD test (*p* ≤ 0.05)

Drought stress increased the antioxidant enzyme activity compared with well water control. However, melatonin application dramatically increased the activity of SOD, CAT, POD, and APX compared to untreated plants. Superoxide dismutase activity (SOD) showed a substantial increase with an increasing the melatonin concentrations for an instant. The results showed that melatonin MT2 treatment increased the SOD activity by 31.64% compared with CKD under drought stress conditions (Fig. 7). The results presented that POD and APX activity have the same increasing trend as that of SOD. Melatonin treatment MT2 increased the POD and APX activity by 46.61 and 101.35% as compared with CKD. A further increase in melatonin concentration slightly decreased SOD, POD, and APX activity, but the treatments MT2 and MT3 were statically not significant. While CAT activity was improved by melatonin treatment, first increasing with rising melatonin concentration and then markedly decreases with higher concentration. The CAT activity showed an increasing trend in melatonin concentration and reached a maximum value under treatment MT2 by 53.18% compared with the CKD.

## Discussion

Drought stress is one of the most destructive abiotic stresses to plants, limiting their physiological, biochemical functions, growth, and biomass production [16]. Melatonin is a master plant growth regulator (PGR) that helps to boost up crop growth and productivity under abiotic stress conditions [1, 7, 16]. However, in this study, exogenous melatonin application decreased the intensity of drought-induced growth inhibition in maize seedlings. The melatonin-treated plants had larger stem diameters, more root biomass, denser roots with greater root length, root shoot ratio, and total dry matter than the control plants (Table 1). A previous study revealed that an increase in biomass accumulation and growth attributes is endorsed to the improved carbon assimilation due to enhanced photosynthetic capacity of the treated plants [7, 16]. As a result, our research indicated that melatonin treatment improved seedling adaptability to drought stress by alleviating the drought-induced suppression of growth characteristics. In addition, we found that the root-drenching method was more effective in improving drought resistance. Various other studies have also shown the stimulatory effect of melatonin on growth and development, in compliance with the current research work evaluated that increased shoot and root weight, fresh and dry biomass of root, stomatal aperture, RWC, protein, soluble sugar, and chlorophyll pigments composition [19, 20]. Melatonin-induced root development is regulated by auxin-modulated physiological processes, affecting water uptake and initiating irreversible cell wall extension [7, 21, 22]. Plant growth regulators are necessary for crop growth and development under environmental stress conditions. The value-added root increases source appears to be melatonin-mediated enhanced adaptability of treated plants for more effective soil water acquisition and nutrients and transport to plants aerial parts. Therefore, our studies show that all plant growth parameters significantly deteriorated in the plants subjected to drought stress (Table 1). These effects may be due to a decrease in photosynthesis, increased evapotranspiration, decreased cell turgidity, limited assimilation of CO_2_ due to stomatal closure, and finally inhibited cell division under drought stress [23]. Melatonin has been implicated in solid evidence as a growth promoter that also increases plant resistance to abiotic stress [24]. While many stressors inhibit plant growth by affecting multiple physiological processes, photosynthesis is most strongly affected by changes in biomass production, which may be due to reduced assimilate requirements [25]. The higher assimilation of carbon corroborates the improvement in biomass production and growth characteristics due to the increased photosynthetic capacity of melatonin-treated plants. Our studies have shown that melatonin application effectively enhanced the adaptability to drought stress by increasing the suppression of drought-induced growth traits of maize seedlings. Previous studies have demonstrated that melatonin treatment alleviates abiotic stress and positively affects growth and development in various plants [17, 26, 27].

Melatonin regulates plant physiology and improves photosynthetic pigments [28, 29]. Chlorophyll is an important pigment for photosynthesis and is involved in the absorption and transmission of light energy [7]. Carotenoids are a form of pigment that acts as a photo protectant and a defensive valve, releasing excess energy before the damage of plant cells [17]. Our results showed that melatonin application reduces the adverse effects of drought stress, improving chlorophyll and carotenoid content compared to untreated control plants under drought stress (Fig. 2). These findings revealed that an optimum melatonin concentration in leaves increased chlorophyll pigment biosynthesis and reduced drought-induced decomposition. This increase in chlorophyll contents is partly contributed to improved photosynthetic gas exchange [7, 20]. These results imply that melatonin has postulated to delay chlorophyll degradation and enhance photosynthetic capacity in crops under abiotic stress conditions [26]. Drought stress causes a significant reduction in leaf area and the degradation of photosynthetic pigments, both of which have a direct impact on plant growth, influencing and minimizing photosynthesis [1, 30]. Melatonin application retains chlorophyll pigments and enhances photosynthesis in plants under drought stress conditions [14]. Our research has shown that the melatonin treatment alleviated the adverse effects of drought stress by increasing the chlorophyll pigments and photosynthetic rate of maize seedling compared with untreated drought stress control plants. One of the primary plant responses to drought stress is stomatal closure to minimize water loss, associated with a severe reduction in stomatal conductance and, subsequently, stomatal limitation of photosynthesis [31]. However, previous studies have shown that an appropriate dose of melatonin application improves stomatal function by stimulating plants to reopen their stomata under water deficit conditions, suggesting that melatonin plays a positive role in stomatal regulation [26, 32, 33]. Our experiment found that the net photosynthetic rate in the untreated plant was significantly lower under drought stress conditions, probably due to stomatal limitation. However, it has been shown that melatonin treatments increase the net photosynthetic rate and stomatal conductance, allowing the RWC of the leaves to increase simultaneously. Consequently, the reduction of stomatal limitation by melatonin contributed to an increase in the net photosynthetic rate under drought stress. Our results are consistent with previous studies that have recorded a comparable increase in photosynthetic rate and stomatal conductance of melatonin-treated wheat seedlings grown under stress conditions [14, 34]. However, exogenous melatonin treatments also contributed by increasing the transpiration rate, likely motivated by improving the stomatal conductance to maintain stable photosynthesis under drought stress conditions.

Leaf stomata are recognized as the primary pores for controlling the transport of CO_2_ and water vapor. However, certain abiotic variables such as atmospheric CO_2_ concentration, water state, temperature, and light can affect these functions [35]. The elimination of transpiration via the closure of stomata is one of the key strategies for plants to boost water status. A decrease in the photosynthesis rate accompanied this method by preventing CO_2_ from entering mesophyll cells [36]. CO_2_ in plant cells directly decreases water stress by reducing diffusion through stomata and reducing CO_2_ in mesophyll by improving leaf carbon metabolism and photochemistry [37, 38]. Photosynthetic rate limitation can be caused by both stomata and non-stomata effects depending on the intensification and crop species. In our research, we investigated stomatal traits by using SEM; we found that stomatal length, stomatal width, stomatal area, and the number of pores were reduced by drought stress compared to control and melatonin treated plants (Fig. 5 & 6). These findings may be due to the accumulation of abscisic acid under drought stress, promoting stomatal closure to prevent water loss from leaf evapotranspiration under drought stress [39, 40].

Plants also adopt a potential strategy to adjust stress conditions by preserving osmotic adaptation by developing substances known as osmolytes with low molecular weight, including soluble sugar, proline, soluble protein, etc. [40, 41]. Proline contributes by quenching singlet oxygen and scavenge OH^•^ radicals to protect turgor cells membrane, DNA and protein, from ROS-induced damage under abiotic stress conditions [42, 43]. Our findings exhibited that the soluble sugar, proline, and soluble protein content of melatonin-treated plants increased significantly under drought stress conditions compared with well water control, representing the possible effectiveness of melatonin in dealing effectively with drought. Previous research results also found that proline accumulation increased under water deficit conditions. A proper amount of exogenous melatonin application (*citrus aurantium* L. and *B. napus* L.) seedling has sustainably increased solutes concentration under abiotic stress [41]. However, we hypothesized that exogenous melatonin stimulated osmotic solvents production, which enhanced the osmotic adaptability of water in plant cells and maintained stomatal control movement by intercepting ROS under drought stress conditions.

Oxidative damage due to excessive accumulation of ROS in plant cells is the primary consequence of environmental stresses [44]. Despite a well-established role of ROS in stress signaling, excessive ROS accumulation could induce oxidative stress [42]. Our studies observed that the H_2_O_2_, O_2,_ and MDA increased under water deficit conditions; however, the exogenous melatonin treatments significantly reduced the MDA content compared to CKD. Higher antioxidant enzyme activity has been linked to lower ROS accumulation in melatonin-treated plants [45]. Plants have evolved an essential antioxidant defense mechanism to deal with oxidative damage, including SOD, POD, CAT, and APX [15].

**Fig. 8:**
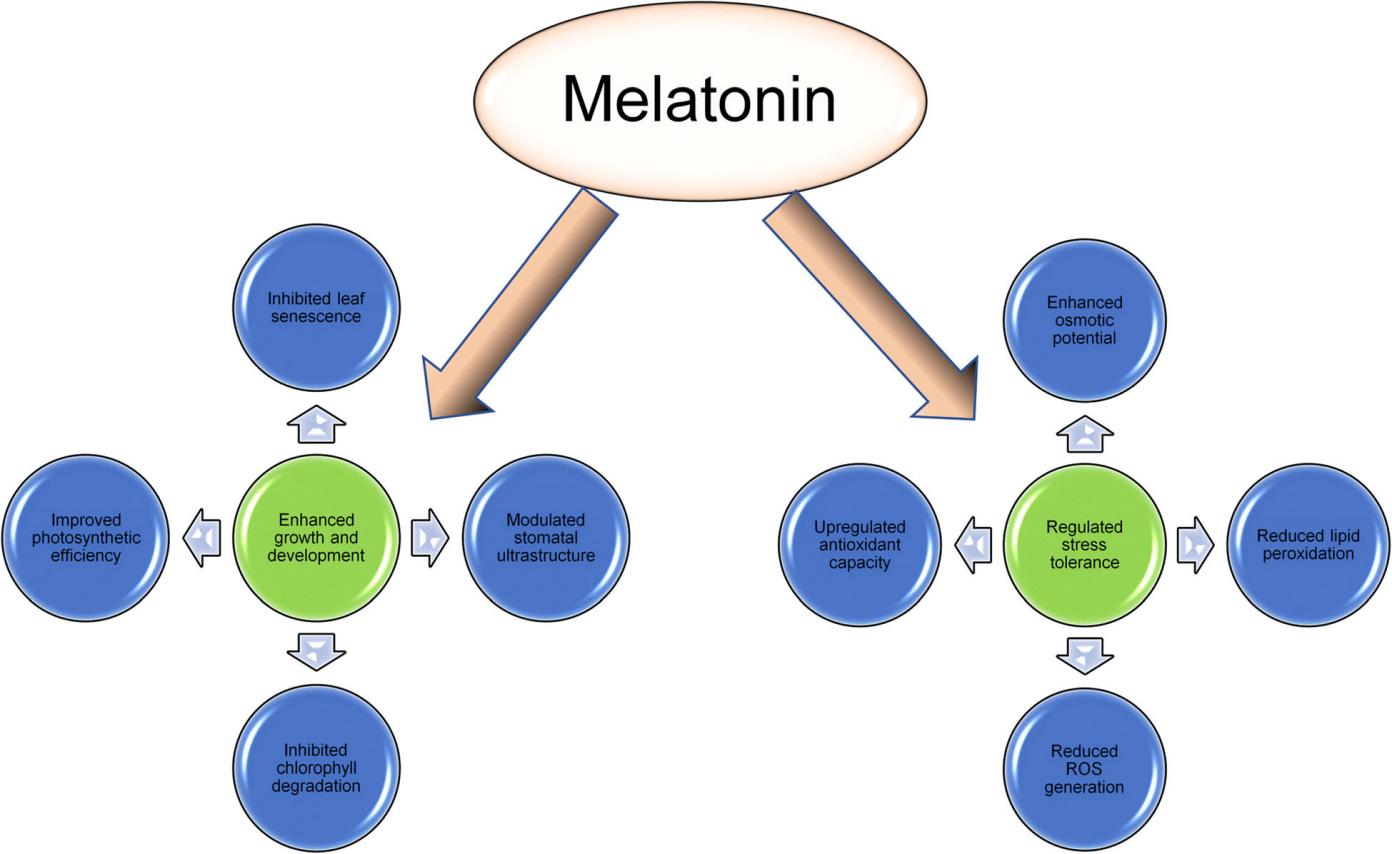
Diagrammatic sketch explaining the action of melatonin on various responses of plants under drought conditions

In contrast to the regulation, all enzymatic antioxidants enzyme activities in our experiment increased in melatonin-treated plants under drought stress compared to well water control. Exogenous melatonin application enhanced the antioxidant enzyme activities including SOD, POD, CAT, and APX that protect plants from ROS induce oxidative damage mediated by melatonin allowed to survive under drought stress conditions. SOD is a central enzyme that controls the O_2_ status and is involved in the main cell defense process [46]. In addition, H_2_O_2_ accumulation was controlled by CAT and APX and reduced to H_2_O [47]. Several studies have demonstrated melatonin’s effectiveness in promoting plant survival and growth by enhancing protective ROS scavenging mechanisms under abiotic stress conditions [33, 48]. Our results suggest that melatonin application reduces harmful drought effects on maize seedlings and improves tolerance to drought stress. Therefore, based on our current research results, the conclusions of previous studies showed that the mitigating impact of melatonin is closely linked to its method of application and acceptable concentrations. These results provide evidence of melatonin physiological function and serve as a forum for future applications in agricultural under drought stress conditions. Fig. 8 summarize the action of melatonin on various responses of maize under drought stress condition.

## Conclusions

The present research work concludes that the exogenous application of melatonin could be an effective approach to improving maize seedling’s resistance to drought stress. The application of melatonin as a soil drenching may enhance enzymatic and non-enzymatic antioxidant activities. This has mainly contributed to improving the antioxidant defense mechanism of maize seedlings, minimizing the oxidative damage caused by drought, as shown by significantly decreasing MDA and ROS accumulation. Also, by osmotic modification, exogenous application of melatonin simulated drought stress by enhancing the levels of osmo-protectants. Melatonin controls root growth to absorb moisture from greater depth in the soil and thus promoted stomatal behavior resulting in an increased net photosynthesis rate in maize seedlings under drought stress conditions. Our research results show the beneficial effects of the melatonin-soil drenching at an appropriate concentration (100 μM) on maize seedling under drought stress conditions. Keeping in view, the morphological, physiological, biochemical, stomatal features from the present study could be employed as an effective approach in many crops and will be useful in elevating drought stress.

## Methods

### Plant material and experimental design

Seeds of hybrid Maize (Wanchuan-1306), the most commonly grown variety in the southern areas of China was used in the current experiment. The seed were obtained from Guangxi Wanchuan seed Industry Co. Ltd., China. The selected seeds permission was granted from the respective authority. The pot experiment was conducted in the glass-shed (net house) of Guangxi University, Nanning, Guangxi, China. The healthy hybrid maize seeds were selected, and five seeds were planted in each pot. The pots size was (32.5 cm diameter and 29 cm height), with 20 kg of soil mixture from arable topsoil was added to each pot. The pots were arranged in a randomized complete block design (RCBD) under natural light in the glass shed. In the early growth stage of maize seedlings, the soil moisture contents were maintained at normal 80–85% field capacity (F.C). A progressive drought was subsequently enforced during the experiment by withholding watering (40–45% F.C), based on daily measurements of pot weight from the five to six-leaf stage. After starting drought stress, the melatonin having different concentrations was applied as soil drenching at 250 mL solution to each pot for three consecutive days at the V-8 stage of maize seedling. The treatments designated for this study include 1. Melatonin 0 μM + Control well water condition (CKW), 2. Melatonin 0 μM + Control drought stress (CKD), 3. Melatonin 50 μM + drought stress (MT1), 4. Melatonin 100 μM + drought stress (MT2), 5. Melatonin 150 μM + drought stress (MT3). All sampling and measurements for different tests, such as leaf ultrastructure, morphological, physiological, and biochemical characteristics, were performed after one week of treatment at the V-9 stage. Three plants (biological replicates) were immediately processed or stored at − 80 °C for further analysis from each treatment.

### Sampling and measurements

#### Determination of plant growth and RWC (%)

Plants from each group treatment were sampled and divided into roots, shoots, and leaves to assess fresh and dry biomass. Root length and diameter were calculated by root image analysis using WinRHIZO 2003a software (Regent Instruments, Québec, Canada). A tape meter was used to measure the plant height and leaf area to detect growth changes. The plant samples were then dried in an oven at 75 °C until constant weight and its dry weight (DW) was determined.

The technique of Su et al. [49] was used to determine the RWC of leaves during the drought stress condition. The fresh weight (FW) of the ninth leaf was measured immediately after harvesting the sample, and then the leaf was placed in deionized water for 24 h, and then the turgid weight (TW) was noted. To obtain the DW, the leaf samples were then oven-dried at 75 °C for 72 h.

The following formula was used to determine the RWC (%);

$$ \mathrm{RWC}=\frac{\left( FW- DW\right)}{\left( TW- DW\right)}\times 100 $$

#### Gas exchange parameters and pigment content

A portable infrared gas analyzer photosynthetic system LI-6800XT (LI-COR, Biosciences, Lincoln, USA) was used to evaluate the net photosynthetic rate, intercellular CO_2_, stomatal conductance, and transpiration rate on a sunny day between the 10:00 and 12:00 pm. Each treatment was repeated three times. The method of Arnon [50] was used for the determination of chlorophyll pigments. Fresh leaf samples 0.1 g were placed in a test tube, 10 mL of 80% cold acetone was added to all test tubes and placed in the dark overnight until the leaf samples had discolored entirely. The samples were then centrifuged, and absorbance of the supernatants was measured at 645, 663, and 440 nm by using a UV-spectrophotometer for the determination of chlorophyll a, b, and carotenoid contents.

#### Scanning electron microscopy (SEM) analysis

The imaging of leaf stomata was done with the help of SEM. Five youngest fully open middle parts of leaves (1 mm) were collected from each treatment. The samples were immediately fixed with a 4% glutaraldehyde solution in 0.1 M phosphate-buffered saline (PBS; pH 6.8). Samples were dehydrated in a graded ethanol series, vacuum dried, and gilded after rinsing five times with PBS for 5, 10, 15, 20, and 30 min [51]. After that, samples were washed with serially diluted ethanol, then subsequently; rinsed twice with isoamyl acetate, and were freeze-dried. The fragments were firmly fixed using double-sided tape on stubs, and sputter-coated was done using gold [51]. The samples were analyzed with a JEOLJSM-6390LV Scanning Electron Microscope. Image-Pro Plus 6.0 software was used to measure the clear stomata from each slice, measure the stomatal length (μm), stomatal width (μm), stomatal area (μm^2^), and count the number of stomata.

#### Determination of soluble sugar and proline content

Soluble sugar content was determined by using the Anthrone method, according to Shi et al. [52]. 0.2 g fresh leaf samples were homogenized with 5 mL ethanol and centrifuged for 10 min at 3500×g. The 3 mL of anthrone reagent was reacted with 0.1 mL of supernatant (150 mg of anthrone + 100 mL of concentrated H_2_SO_4_). The mixture was incubated in a boiling water bath for 10 min, after which the absorbance was recorded at 630 nm using a UV-spectrophotometer. The leaf proline content was determined by the method to describe by Tiwari et al. [53]. The 0.5 g fresh leaf sample was homogenized in 10 mL aqueous sulfosalicylic acid (3%) and centrifuged at 10,000×g for 15 min. 2 mL of the extract was mixed with 2 mL glacial acetic acid and 4 mL toluene. The absorbance was determined at 520 nm by UV-spectrophotometer, using toluene for a blank.

#### Measurement of soluble protein, MDA and ROS

Soluble protein content was estimated by using the Coomassie Brilliant Blue G-250 solution method according to Zhao et al. [54]. 0.5 g fresh leaf samples were homogenized in 10 ML phosphate buffer (pH 7.0) and centrifuged at 15,000×g for 20 min. The absorption of the prepared solution by a spectrophotometer against a blank reagent was recorded at 595 nm. The soluble protein concentration was expressed as mg g^− 1^ FW. MDA as an end product of lipid peroxidation as measured by Heath et al. [55]. A fresh leaf sample 0.5 g was extracted with 10 mL of ethanol and centrifuged for 10 min at 25 °C (4000×g). A 20% (trichloroacetic acid) and 0.65% (thiobarbituric acid) reaction mixture (2 mL) was added to extracts (1 mL) in a test tube. The samples were heated at 100 °C for 20 min in a water bath and at 10,000×g after cooling for 5 min. At 440, 532, and 600 nm, the absorbance of the samples was reported, and the MDA content was expressed in μmol g^− 1^ FW.

For the determination of H_2_O_2_, the method described by Zhang et al. [56] was used; by adding 200 mL of enzyme extract to the reaction solution containing a BPS (2.5 mM, pH 7.0) phosphate buffer and replacing H_2_O_2_ with potassium iodide (500 mM). Incubate the reaction mixture at 25 °C for 1 h, and H_2_O_2_ is used as a standard, and the absorbance was measured at 390 nm by using a UV-spectrophotometer. Following the protocol with a slight modification of Zhang et al. [56], the rate of O_2_ generation was calculated. A fresh leaf sample of 0.2 g was extracted in 1 mL (BPS 50 mM, pH 7.8) and then centrifuged at 10,000×g. A reaction mixture of sulfanilamide (17 mM) and naphthalene diamine hydrochloride was then applied to 1 mL of supernatant (7 mM). The resulting mixture was incubated at 37 °C for 10 min, and 3 mL of ether was added to each tube. Samples were centrifuged at 24 °C for 5 min (5000×g), and absorbance was determined using a UV-spectrophotometer at 540 nm.

#### Antioxidant enzymes (SOD, POD, CAT & APX) extraction assay

Fresh leaf sample (0.2 g) was harvested by removing midrib, weighed, and washed with distilled water and then homogenized with a 5 ml chilled sodium phosphate buffer mortar and pestle (50 mM, pH 7.8). The homogenates were centrifuged at 15,000×g for 15 min at 4 °C. The supernatant was kept at 4 °C and used for assays of SOD, CAT, POD, and APX activities and expressed as U mg^− 1^ min^− 1^ FW.

The activity of SOD was determined according to the protocol of Giannopolitis et al. [57] after photoreduction of NBT (Nitroblue Tetrazolium). Enzyme extract (20 μL) was added to a SOD reaction mixture of 0.3 mL methionine (13 mM), 1.5 mL phosphate buffer (50 mM, pH 7.8), 0.3 mL EDTA-Na2 (0.1 mM), 0.3 mL NBT (750 mM), 0.3 mL riboflavin (20 M), and 0.3 mL distilled water. A spectrophotometer was used to record the absorbance of the solutions at 560 nm.

The CAT activity procedure was described by Wang et al. [58]. The CAT reaction mixture consisting of 2 mL phosphate buffer (50 mM, pH 7.0 with 0.1 mM EDTA) and 0.5 mL H_2_O_2_ was added to the enzyme extract (20 μL). The reaction was started after the addition of H_2_O_2,_ and a spectrophotometer observed the decomposition rate of H_2_O_2_ at 240 nm.

POD activity was calculated according to Ekemkci et al. [59] with little modifications. Enzyme extract (20 μL) was added to a POD reaction mixture of 1.5 mL BPS (50 mM, pH 7.8), 0.5 mL guaiacol (50 mM), 0.5 mL H_2_O_2_ (200 mM), and 0.5 mL water. Using a spectrophotometer to measure POD enzyme activity, increases in absorbance due to guaiacol oxidation at 470 nm were observed.

APX activity was tested according to the method of Nakano et al. [60]. To 3 mL of reaction mixtures containing BPS (50 mM, pH 7.0), 0.2 mM EDTA 0.5 mM ascorbic acid, and an amount of 0.1 mL enzyme extract were added 0.1 mM H_2_O_2_. The absorbance was measured immediately at 240 nm with a UV-spectrophotometer.

### Statistical analysis

Statistical analysis was done using Statistic 8.1 software to evaluate the obtained data; the Least Significance Difference (LSD) test was used to equate the means at the significance level of *p* ≤ 0.05. For each treatment, means were expressed as ± standard deviation (SD) of triplicates (*n* = 3). Image-Pro Plus 6.0 software was used to measure the clear stomata from each slice, measure the stomatal width (μM), stomatal length (μM), stomatal area (μM^2^), and count the number of stomata.

## Data Availability

The datasets used and/or analyzed during the current study available from the corresponding author on reasonable request.

## Abbreviations

ROS: Reactive oxygen species
SOD: Superoxide dismutase
POD: Peroxidase
CAT: Catalase
APX: Ascorbate peroxidase
AsA-GSH: Ascorbate glutathione
PGR: Plant growth regulator
MDA: Malondialdehyde
GSH: Glutathione
ASA: Ascorbate
O_2_: Superoxide
OH: Hydroxyl ion
H_2_O_2_: Hydrogen peroxide
F.C: Field capacity
CKW: Control well water condition
CKD: Control drought stress
MT: Melatonin treatments
RWC: Relative water content
FW: Fresh weight
TW: Turgid weight
DW: Dry weight
SEM: Scanning electron microscopy
LSD: Least significant difference
IAA: Indole acetic acid
